# Supplementary material for: Neutralizing antibodies from prior exposure to dengue virus negatively correlate with viremia on re-infection
Source: Commun Med (Lond). 2023 Oct 19;3:148. doi: 10.1038/s43856-023-00378-7 (PMC10587183; doi:10.1038/s43856-023-00378-7)
Supplement: Supplementary file 3 — Supplementary Information [file 43856_2023_378_MOESM3_ESM.pdf]

SUPPLEMENTARY INFORMATION

SUPPLEMENTARY METHODS

Viral RNA extraction and cDNA synthesis

DENV RNA was extracted from culture supernatants of C6/36 cells infected with DENV-1, DENV-2, DENV-3 and DENV-4 serotypes using QIAmp viral RNA Kit (QIAGEN) as per the manufacturer's protocol. RNA was eluted in 50 µL of AVE buffer as provided in the kit. The extracted dengue viral RNA was reverse transcribed to synthesize cDNA using Superscript™ IV Reverse Transcriptase (SS IV RT) (Invitrogen Bio services India Pvt. Ltd.). Briefly, 11 µL of the extracted RNA was added to the 1 µL of 0.2 µM reverse primer pool (prepared by adding all the reverse primer in equal volume in a 0.2 ml tube) specific for the serotypes, 1 µL of 10 mM dNTP mix and incubated at 60 °C for 10 min to allow the primers to anneal to the target efficiently. To synthesize the first strand, the above product was mixed with the 4 µL of 5x superscript IV buffer, 1 µL of 10 mM DTT, 1 µL of RNase out RNase inhibitor and 1 µL of SS IV RT enzyme. The total reaction of 20 µL was subjected to reverse transcription at 42°C for 30 min followed by inactivation at 85 °C for 5 sec.

Whole genome amplification

Dengue whole genome was PCR amplified by using serotype specific overlapping primers (Supplementary data 1). The PCR were performed in a reaction mixture of 20 µL containing 2 µL of 10X Standard Taq reaction buffer (B9014S, New England Biolabs), 0.8 µL of dNTPs (10 mM), 0.4 µL of forward Primer (200 µM), 0.4 µL of reverse primer (200 µM) 0.1 µL of Taq DNA polymerase (M0273L, NEB) and 1 µL of cDNA as template. The PCR reaction involved one cycle of initial denaturation at 94 °C for 3 min followed by 30 cycles of denaturation at 94°C for 30 sec, annealing at 60 °C for 1 min 30 sec, extension at 68 °C for 1 min 30 sec and one cycle of final extension at 68 °C for 5 min. The amplified products were purified from 1 % agarose gel using PCR Clean-up system (NucleoSpin Gel and PCR Clean-up, Macherey-Nagel GmbH & Co. KG, Germany).

Complete genome sequencing

Full genome sequencing of viral genomes was sequenced using MinION Mk1C platform. Briefly, the concentration of the PCR amplicons was measured using NanoDrop™ 2000 Spectrophotometer (Thermo Scientific™, USA) and the integrity of DNA was observed on agarose gel electrophoresis. The samples that passed quality assessment with optimal yield and concentration, were deemed suitable for nanopore library preparation. A total of 5 µl of each amplicon was used for fragmentation and barcode ligation (SQK-RBK110.96). After barcode ligation all samples were pooled and cleaned with 1X AMPure beads (Beckmann Coulter, USA). Cleaned pool was qubit quantified and 600 ng of the pooled sample was attached with sequencing adaptor (RAPF) by enzyme free mechanism. Sequencing was performed on Mk1C device (Oxford Nanopore Technologies, Oxford, UK) using SpotON flow cell R9.4 (FLO-MIN106) in a 48 hrs sequencing protocol. Nanopore raw reads ('fast5' format) were base-called ('fastq' format) and de-multiplexed using Guppy v2.3.4. The raw nanopore reads were processed for adapters trimming using Porechop-v0.2.31. The good quality adapter free reads were mapped against specific reference viral sequences using Minimap-v2.2 tool. Each specific DENV serotype samples were mapped against specific reference viral genomes. The alignment percentage across samples was in the range of ~76-78 %. Next, the alignment data was further processed using Bcftools-v1.123 and Samtools-v1.94 pipeline to generate consensus sequences and predict variants. The raw variants identified were filtered based on minimum quality score of 30 and read depth of 20 to obtain the filtered variants. Finally, the filtered variants were

annotated using snpEff-v3.3h5 tool to retrieve protein level change information. Complete genome sequences were submitted to GenBank.

Phylogenetic analysis

The complete coding sequence of selected sequences were used for phylogenetic analysis as per recent reports[1]. Multiple sequence analysis was performed using MUSCLE (v.3.8.425) in Aliview(v 1.28). Maximum likelihood trees were inferred using iqtree (v2.2.0), with ultrafast bootstrap (1000 replicates). Trees were visualized using Figtree (v1.4.4), and rooted by midpoint. Bootstrap support values are shown at the nodes and tips are labelled as Genbank ID, genotype, country and collection year of the sequence.

SUPPLEMENTARY TABLES

Supplementary Table S1: Sequences of primers and probe used in quantitative RT-PCR for detection of DENV RNA

S. No.	Primer / Probe	Primer / Probe sequence (5' - 3')
1	Den-UTR forward Primer	AACAGCATATTGACGCTG
2	Den-UTR reverse Primer	CTGTGCCTGGAATGATG
3	Den-UTR Probe	[6~FAM] ACCAGAGATCCTGCTGTC [Tamra~Q]

Supplementary Table S2: Sequences of primers and probe used in quantitative RT-PCR for detection of DENV RNA

S. No.	Primer name	Primer sequence sequence (5' - 3')
1	D1	TCAATATGCTGAAACGCGCGAGAAACCG
2	D2	TTGCACCAACAGTCAATGTCTTCAGGTTC
3	TS1	CGTCTCAGTGATCCGGGGG
4	TS2	CGCCACAAGGGCCATGAACAG
5	TS3	TAACATCATCATGAGACAGAGC
6	TS4	CTCTGTTGTCTTAAACAAGAGA

Supplementary Table S3: **DENV, ZIKV, JEV and WNV $FRNT_{50}$ titers in serum of dengue patients (n=76)**

Virus used for neutralization	DENV-1	DENV-2	DENV-3	DENV-4	ZIKV	JEV	WNV
Geometric mean (95% CI)	453 (310-661)	634 (414-972)	259 (181-369)	199 (134-294)	90 (62-132)	26 (19-35)	53 (37-77)

DENV-1: Dengue virus-1, DENV-2: Dengue virus-2, DENV-3: Dengue virus-3, DENV-4, Dengue virus-4, ZIKV: Zika virus, JEV: Japanese encephalitis virus, WNV: West Nile virus.

Supplementary Table S4: **Samples with $FRNT_{50}$ titers for only DENV**

Virus used for neutralization (n=12)	DENV-1	DENV-2	DENV-3	DENV-4
Geometric mean	288.6	310.5	102.1	59.8

Lower 95% CI of geo. mean	150.2	113.3	53.0	33.1
Upper 95% CI of geo. mean	554.4	851.4	196.9	108.3

DENV-1: Dengue virus-1, DENV-2: Dengue virus-2, DENV-3: Dengue virus-3, DENV-4, Dengue virus-4.

Supplementary Table S5: **Samples with $FRNT_{50}$ titers to DENV and ZIKV**

Virus used for neutralization (n=6)	DENV-1	DENV-2	DENV-3	DENV-4	ZIKV
Geometric mean	500.7	261.3	352.5	87.3	138.2
Lower 95% CI of geo. mean	131.4	66.9	150.6	32.9	77.3
Upper 95% CI of geo. mean	1908	1021	825.2	231.7	246.8

DENV-1: Dengue virus-1, DENV-2: Dengue virus-2, DENV-3: Dengue virus-3, DENV-4, Dengue virus-4, ZIKV: Zika virus.

Supplementary Table S6: **Samples with $FRNT_{50}$ titers to DENV and WNV**

Virus used for neutralization (n=8)	DENV-1	DENV-2	DENV-3	DENV-4	WNV
Geometric mean	1334	1132	561.8	438.3	83.38
Lower 95% CI of geo. mean	340.3	269.7	182.7	100.1	23.70
Upper 95% CI of geo. mean	5229	4750	1727	1920	293.4

DENV-1: Dengue virus-1, DENV-2: Dengue virus-2, DENV-3: Dengue virus-3, DENV-4, Dengue virus-4, WNV: West Nile virus.

Supplementary Table S7: **Samples with $FRNT_{50}$ titers to indicated flaviviruses**

Virus used for neutralization (n=16)	DENV-1	DENV-2	DENV-3	DENV-4	ZIKV	WNV
Geometric mean	617.5	1923	622.3	439.1	317.7	68.36
Lower 95% CI of geo. mean	258.8	1048	280.3	216.7	145.7	43.3
Upper 95% CI of geo. mean	1474	3531	1382	889.5	692.5	108.0

DENV-1: Dengue virus-1, DENV-2: Dengue virus-2, DENV-3: Dengue virus-3, DENV-4, Dengue virus-4, ZIKV: Zika virus, WNV: West Nile virus.

Supplementary Table S8: **Samples with $FRNT_{50}$ titers to indicated flaviviruses**

Virus used for neutralization (n=14)	DENV-1	DENV-2	DENV-3	DENV-4	JEV	WNV
Geometric mean	321.3	502.6	145.4	165.0	133.9	159.4

Lower 95% CI of geo. mean	169.5	195.0	61.0	53.8	74.94	65.14
Upper 95% CI of geo. mean	609.0	1295	346.7	506.3	239.2	390.1

DENV-1: Dengue virus-1, DENV-2: Dengue virus-2, DENV-3: Dengue virus-3, DENV-4, Dengue virus-4, JEV: Japanese encephalitis virus, WNV: West Nile virus.

Supplementary Table S9: **Samples with $FRNT_{50}$ titers to all the flaviviruses listed in the table**

Virus used for neutralization (n=13)	DENV-1	DENV-2	DENV-3	DENV-4	ZIKV	WNV	JEV
Geometric mean	1417	2073	667.5	630.2	584.7	233.5	85.75
Lower 95% CI of geo. mean	579.1	763.0	368.1	228.8	241.7	113.2	32.84
Upper 95% CI of geo. mean	3466	5631	1210	1736	1414	481.8	223.9

DENV-1: Dengue virus-1, DENV-2: Dengue virus-2, DENV-3: Dengue virus-3, DENV-4, Dengue virus-4, ZIKV: Zika virus, WNV: West Nile virus, JEV: Japanese encephalitis virus.

SUPPLEMENTARY FIGURE LEGENDS

Supplementary Figure S1: *DENV RNA levels in serotype detected and indeterminate samples.* Total RNA was extracted from serum samples and DENV RNA levels were measured by quantitative RT-PCR. Dengue serotyping was performed by nested PCR. The viral RNA levels in serotype positive (blue) (n=272) and serotype-negative samples (red) (n=140) are compared using Mann-Whitney test. Two-tailed P value is indicated. **** P= <0.0001. LOQ- Limit of quantitation.

Supplementary Figure S2: *Focus reduction neutralization titer (FRNT) assay*. **A**. 96-well plate-based microneutralization assay to detect neutralizing antibodies against all four dengue serotypes was developed in LLCMK2 cells. Representative foci counts and images for Dengue IgG positive serum sample showing neutralization of all four serotypes are shown, shown, DENV-1: grey, DENV-2: pink, DENV-3: green, DENV-4: purple. Final serum dilutions are depicted on the left panel. Virus control shows wells where no serum was added. **B**. Samples negative for dengue antibodies by ELISA were tested for neutralizing antibody titers that reduce the infection by 50% (FRNT50) against DENV to determine the limit of quantitation (LOQ) and background values of the FRNT assay.

Supplementary Figure S3: Maximum Likelihood Phylogenetic tree of DENV1 using coding region sequences (n=48). Nodes as labelled with bootstrap support. The genotypes of virus used in the study and the backbone for the vaccine are highlighted. Genotype I - orange, genotype II - black, genotype III - pink, genotype IV - green and genotype V - blue. Vaccine strain is indicated as green square and the Indian isolate used in the FRNT assay is indicated by pink circle.

Supplementary Figure S4: Maximum Likelihood Phylogenetic tree of DENV2 using coding region sequences (n=30). Nodes as labelled with bootstrap support. The genotypes of virus used in the study and the backbone for the vaccine are highlighted. Cosmopolitan lineage -

pink, Asian-American - dark blue, Asian - green. Vaccine strain is indicated as green square and the Indian isolate used in the FRNT assay is indicated by pink circle.

Supplementary Figure S5: Maximum Likelihood Phylogenetic tree of DENV3 using coding region sequences (n=39). Nodes as labelled with bootstrap support. The genotypes of virus used in the study and the backbone for the vaccine are highlighted. Genotype I - green, genotype II - black, genotype III - pink. Vaccine strain is indicated as green circle and the Indian isolate used in the FRNT assay is indicated by pink square.

Supplementary Figure S6: Maximum Likelihood Phylogenetic tree of DENV4 using coding region sequences (n=24). Nodes as labelled with bootstrap support. The genotypes of virus used in the study and the backbone for the vaccine are highlighted. Genotype I - pink, genotype II - green. Vaccine strain is indicated as green square and the Indian isolate used in the FRNT assay is indicated by pink square.

SUPPLEMENTARY REFERENCES

1. Jagtap S, Pattabiraman C, Sankaradoss A, Krishna S, Roy R. Evolutionary dynamics of dengue virus in India. *PLoS Pathog* 2023; **19**(4): e1010862.

Supplementary Figure S1

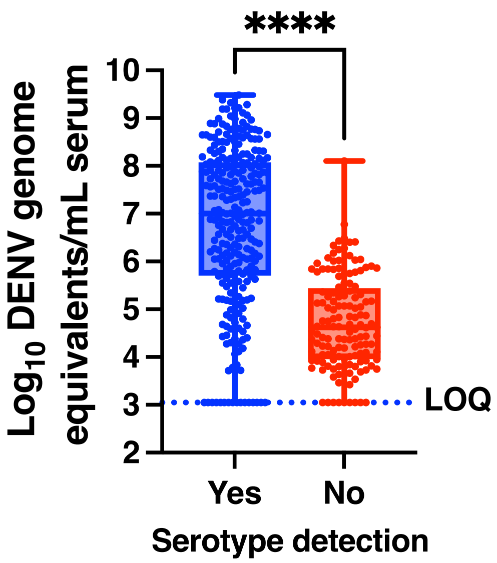

Supplementary Figure S1: *DENV RNA levels in serotype detected and indeterminate samples.* Total RNA was extracted from serum samples and DENV RNA levels were measured by quantitative RT-PCR. Dengue serotyping was performed by nested PCR. The viral RNA levels in serotype positive (blue) (n=272) and serotype negative (red) samples (n=140)are compared using Mann-Whitney test. Two-tailed P value is indicated**.** **** P= <0.0001**.** LOQ- Limit of quantitation.

Supplementary Figure S2

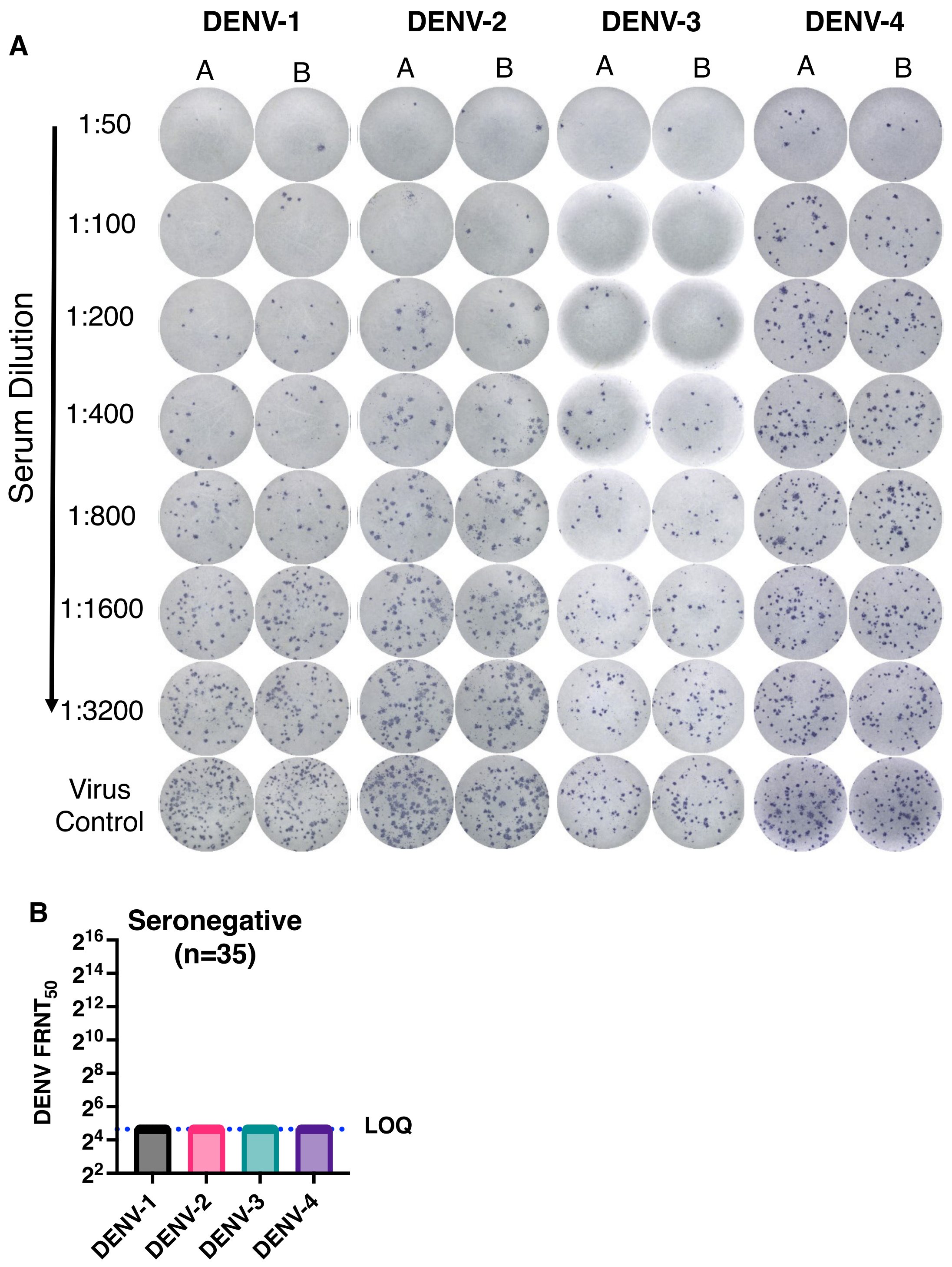

Supplementary Figure S2: *Focus reduction neutralization titer (FRNT) assay.* **A.** 96-well plate-based microneutralization assay to detect neutralizing antibodies against all four dengue serotypes was developed in LLCMK2 cells. Representative foci counts and images for Dengue IgG positive serum sample showing neutralization of all four serotypes are shown, DENV-1: grey, DENV-2: pink, DENV-3: green, DENV-4: purple. Final serum dilutions are depicted on the left panel.Virus control shows wells where no serum was added. **B**. Samples negative for dengue antibodies by ELISA were tested for neutralizing antibody titers that reduce the infection by 50% (FRNT50) against DENV to determine the limit of quantitation (LOQ) and background values of the FRNT assay.

Supplementary Figure S3

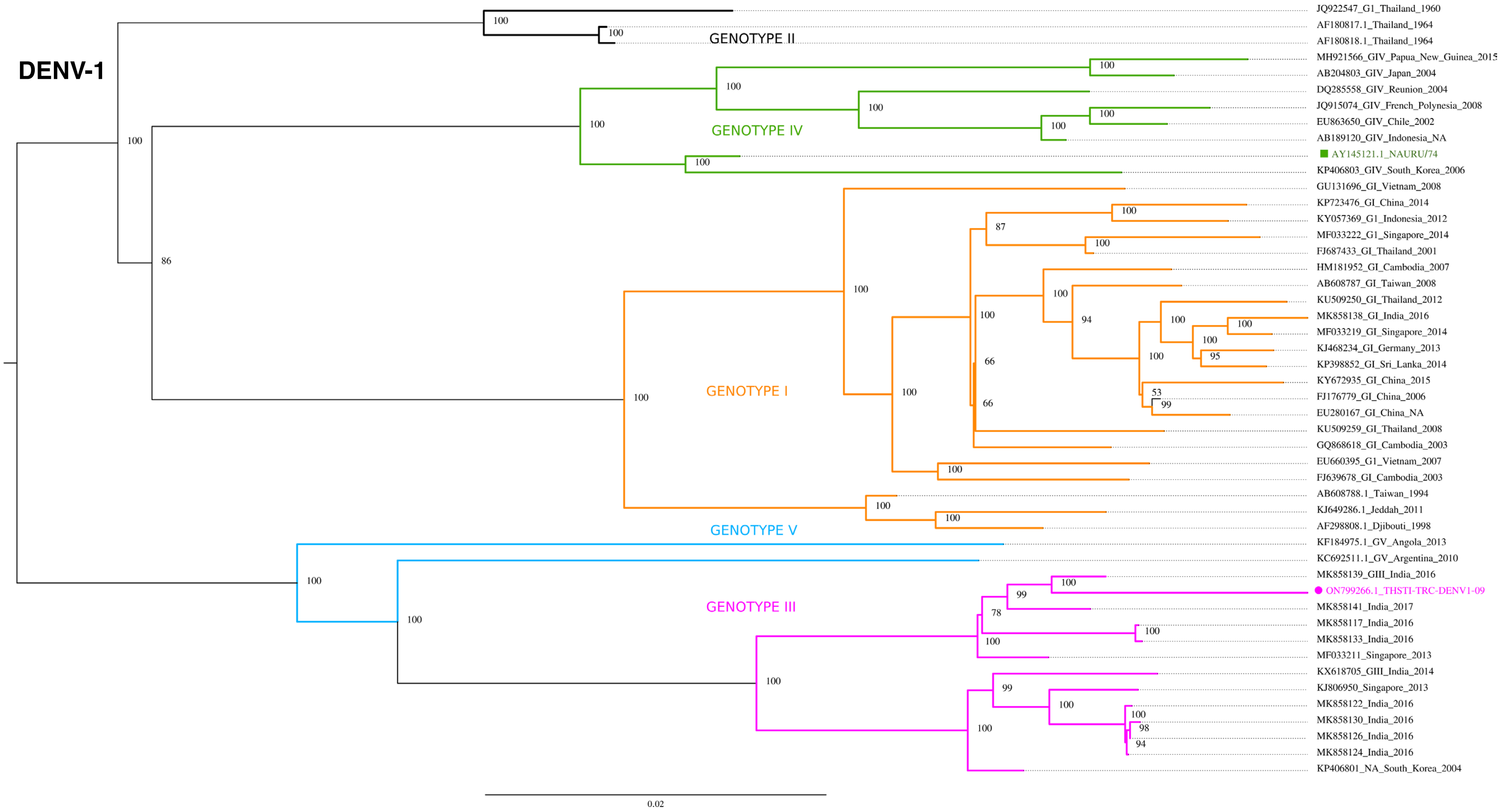

Supplementary Figure S3: Maximum Likelihood Phylogenetic tree of DENV1 using coding region sequences (n=48). Nodes as labelled with bootstrap support. The genotypes of virus used in the study and the backbone for the vaccine are highlighted. Genotype I - orange, genotype II - black, genotype III - pink, genotype IV - green and genotype V - blue. Vaccine strain is indicated as green square and the Indian isolate used in the FRNT assay is indicated by pink circle.

Supplementary Figure S4

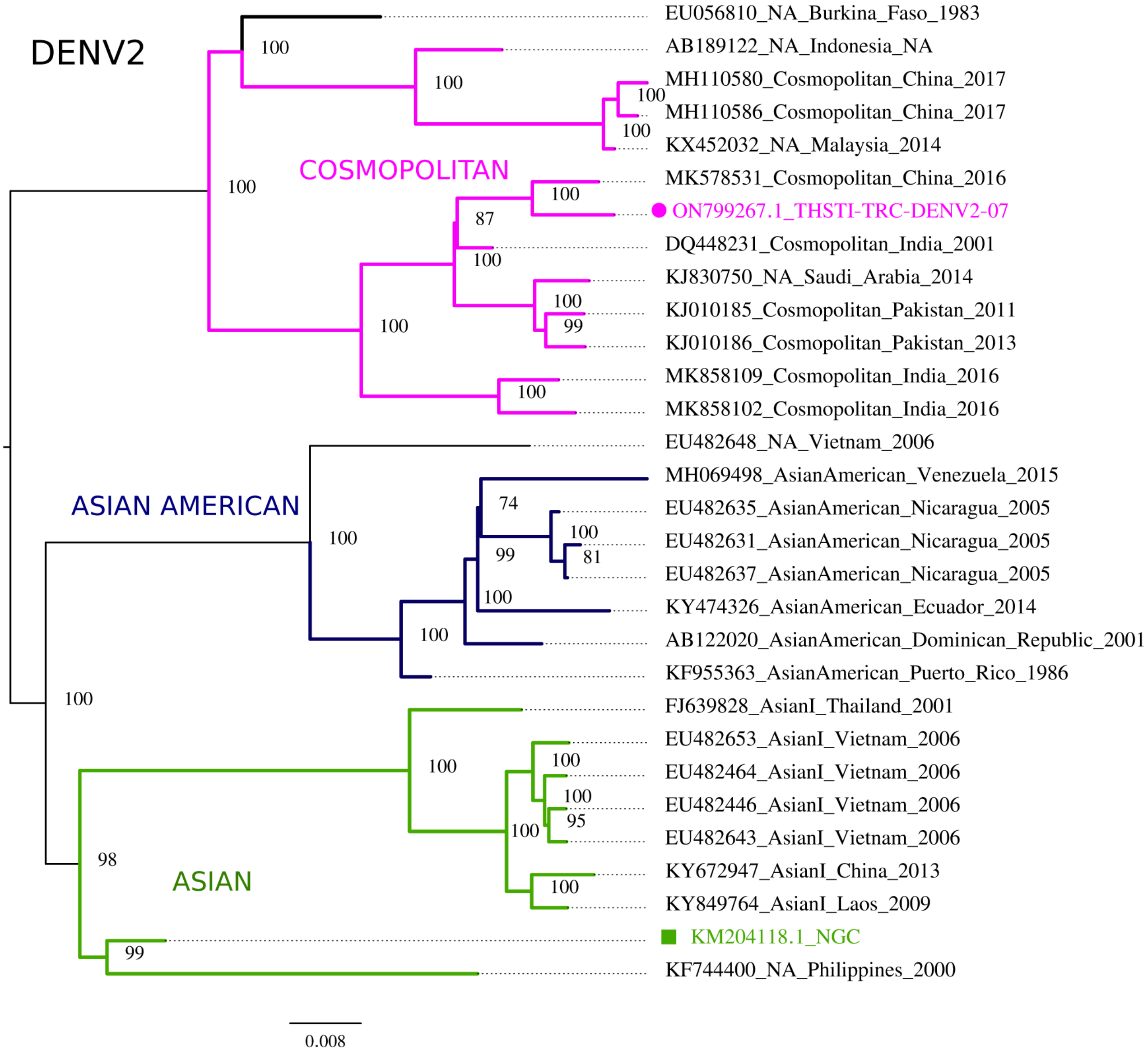

Supplementary Figure S4: Maximum Likelihood Phylogenetic tree of DENV2 using coding region sequences (n=30). Nodes as labelled with bootstrap support. The genotypes of virus used in the study and the backbone for the vaccine are highlighted. Cosmopolitan lineage - pink, Asian-American - dark blue, Asian - green. Vaccine strain is indicated as green square and the Indian isolate used in the FRNT assay is indicated by pink circle.

Supplementary Figure S5

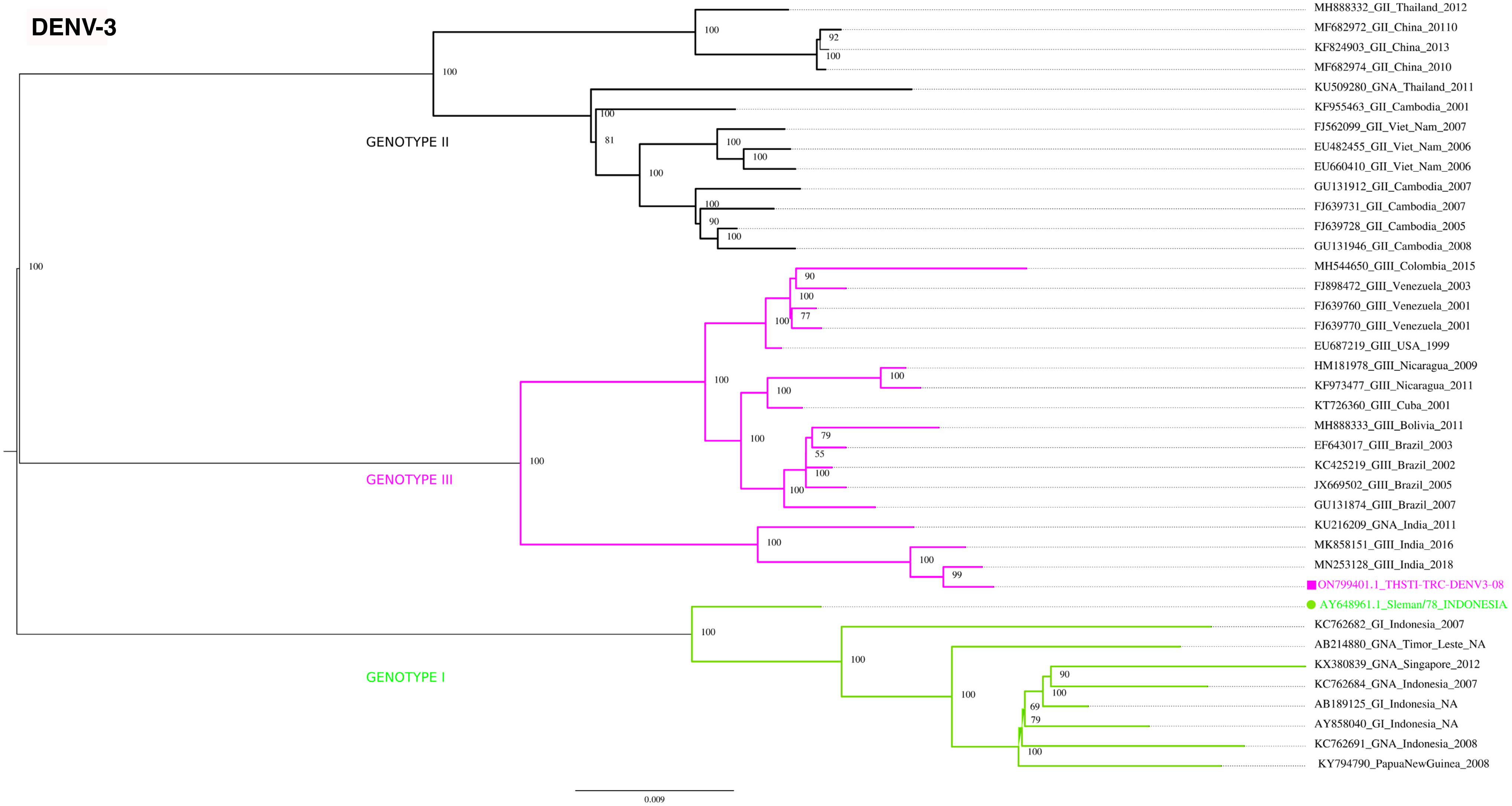

Supplementary Figure S5: Maximum Likelihood Phylogenetic tree of DENV3 using coding region sequences (n=39). Nodes as labelled with bootstrap support. The genotypes of virus used in the study and the backbone for the vaccine are highlighted. Genotype I - green, genotype II - black, genotype III - pink. Vaccine strain is indicated as green circle and the Indian isolate used in the FRNT assay is indicated by pink square.

Supplementary Figure S6

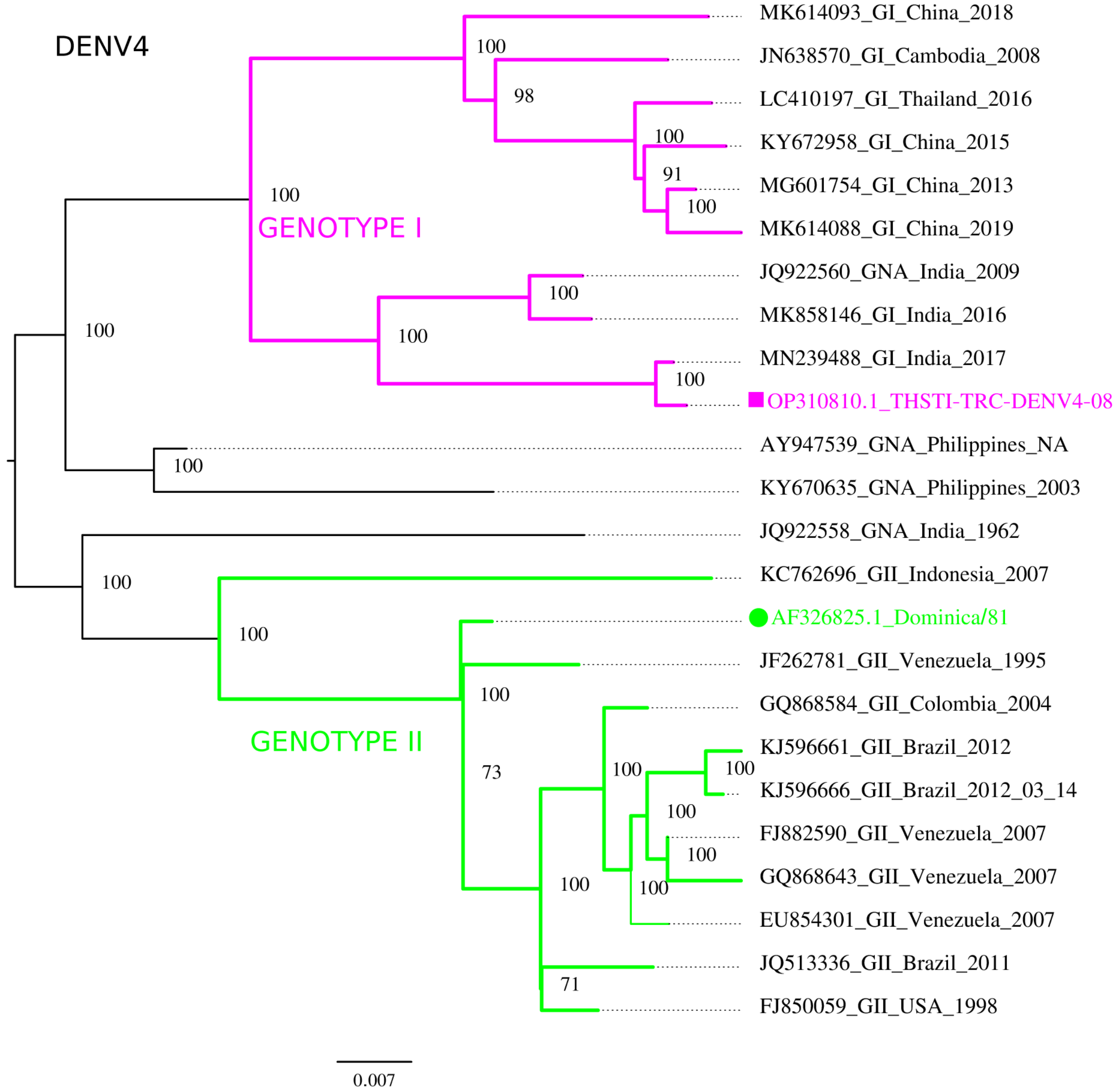

Supplementary Figure S6: Maximum Likelihood Phylogenetic tree of DENV4 using coding region sequences (n=24). Nodes as labelled with bootstrap support. The genotypes of virus used in the study and the backbone for the vaccine are highlighted. Genotype I - pink, genotype II - green. Vaccine strain is indicated as green square and the Indian isolate used in the FRNT assay is indicated by pink square.
