# Supplementary material for: Neutralizing antibodies from prior exposure to dengue virus negatively correlate with viremia on re-infection
Source: Commun Med (Lond). 2023 Oct 19;3:148. doi: 10.1038/s43856-023-00378-7 (PMC10587183; doi:10.1038/s43856-023-00378-7)
Supplement: Supplementary file 4 — Description of Additional Supplementary Files [file 43856_2023_378_MOESM4_ESM.pdf]

Description of Additional Supplementary Files

File Name: Supplementary Data 1
Description: List of primer pairs used for generation of amplicons by PCR for sequencing of Indian DENV strains.

File Name: Supplementary Data 2
Description: Source data of numerical values used to generate the main figures in the manuscript.
