# Supplementary material for: Neutralizing antibodies from prior exposure to dengue virus negatively correlate with viremia on re-infection
Source: Commun Med (Lond). 2023 Oct 19;3:148. doi: 10.1038/s43856-023-00378-7 (PMC10587183; doi:10.1038/s43856-023-00378-7)
Supplement: Supplementary file 6 — Reporting Summary [file 43856_2023_378_MOESM6_ESM.pdf]

nature portfolio

Corresponding author(s): Guruprasad R. Medigeshi

Last updated by author(s): Aug 11, 2023

Reporting Summary

Nature Portfolio wishes to improve the reproducibility of the work that we publish. This form provides structure for consistency and transparency in reporting. For further information on Nature Portfolio policies, see our Editorial Policies and the Editorial Policy Checklist.

Statistics

For all statistical analyses, confirm that the following items are present in the figure legend, table legend, main text, or Methods section.

n/a	Confirmed	
☐	☒	The exact sample size (*n*) for each experimental group/condition, given as a discrete number and unit of measurement
☐	☒	A statement on whether measurements were taken from distinct samples or whether the same sample was measured repeatedly
☐	☒	The statistical test(s) used AND whether they are one- or two-sided<br>*Only common tests should be described solely by name; describe more complex techniques in the Methods section.*
☐	☒	A description of all covariates tested
☐	☒	A description of any assumptions or corrections, such as tests of normality and adjustment for multiple comparisons
☐	☒	A full description of the statistical parameters including central tendency (e.g. means) or other basic estimates (e.g. regression coefficient) AND variation (e.g. standard deviation) or associated estimates of uncertainty (e.g. confidence intervals)
☐	☒	For null hypothesis testing, the test statistic (e.g. *F*, *t*, *r*) with confidence intervals, effect sizes, degrees of freedom and *P* value noted<br>*Give P values as exact values whenever suitable.*
☒	☐	For Bayesian analysis, information on the choice of priors and Markov chain Monte Carlo settings
☒	☐	For hierarchical and complex designs, identification of the appropriate level for tests and full reporting of outcomes
☒	☐	Estimates of effect sizes (e.g. Cohen's *d*, Pearson's *r*), indicating how they were calculated

Our web collection on statistics for biologists contains articles on many of the points above.

Software and code

Policy information about availability of computer code

Data collection	ELISA data were collected using Gen5 (v 3.10) software from BioTek. RT PCR data were collected using Bio-rad CFX Maestro software version 1. Serotyping data were collected using QIAxcel ScreenGel Software version 1.6. All the data were entered in Microsoft Excel v 16.16.27.
Data analysis	Data were analysed and final graphs were prepared using GraphPad Prism (Version 9.5.1) software. FRNT data was calculated by SoftMax Pro GxP software v7.7.1 (Molecular Devices). Multiple sequence analysis was performed using MUSCLE (v.3.8.425) in Aliview(v 1.28). Maximum likelihood trees were inferred using iqtree (v2.2.0). Trees were visualized using Figtree (v1.4.4).

For manuscripts utilizing custom algorithms or software that are central to the research but not yet described in published literature, software must be made available to editors and reviewers. We strongly encourage code deposition in a community repository (e.g. GitHub). See the Nature Portfolio guidelines for submitting code & software for further information.

Data

Policy information about availability of data

All manuscripts must include a data availability statement. This statement should provide the following information, where applicable:

- Accession codes, unique identifiers, or web links for publicly available datasets
- A description of any restrictions on data availability
- For clinical datasets or third party data, please ensure that the statement adheres to our policy

All data generated or analyzed during this study are included in this manuscript. The virus strains used in this study have the following accession numbers:Dengue 1

(Genbank: ON799266), Dengue 2 (Genbank: ON799267), Dengue 3 (Genbank: ON799401) and Dengue 4 (Genbank: OP310810). Dengue International strains, JEV and WNV used in this study was previously reported and the same is cited in the manuscript. Zika virus (MR-766 strain) was obtained from BEI resources (NR-50065). Source data is provided with this paper.

Human research participants

Policy information about studies involving human research participants and Sex and Gender in Research.

Reporting on sex and gender	Leftover samples from a diagnostic lab was used. No personal identifiers or clinical data of the patients were obtained with the samples.
Population characteristics	The median age of subjects was 30 years (Range 0-85 years). No other personal identifiers or clinical data of the patients were obtained with the samples.
Recruitment	Anonymous, leftover samples (n=412) post-dengue diagnosis by NS-1 or IgM ELISA were obtained from a centralized diagnostic lab in the National Capital Region (NCR) of Delhi.
Ethics oversight	The Institutional Ethics Committee for Human Research of Translational Health Science and Technology Institute has exempted this study from review.

Note that full information on the approval of the study protocol must also be provided in the manuscript.

Field-specific reporting

Please select the one below that is the best fit for your research. If you are not sure, read the appropriate sections before making your selection.

☒ Life sciences ☐ Behavioural & social sciences ☐ Ecological, evolutionary & environmental sciences

For a reference copy of the document with all sections, see nature.com/documents/nr-reporting-summary-flat.pdf

Life sciences study design

All studies must disclose on these points even when the disclosure is negative.

Sample size	No sample size calculation was performed. Objective of this study was to estimate the prevalence of dengue antibodies in samples collected for diagnosis of suspected dengue cases during the dengue season of 2018-2019 from the National Capital Region of India. Our study presents a comprehensive overview of binding and neutralizing antibodies for dengue viruses in a cohort of patients with acute febrile illness and shows correlation between pre-existing antibodies, RNAemia and circulating dengue viruses in the National Capital Region of India.
Data exclusions	No data were excluded
Replication	All the assays involved testing the independent clinical samples (n=412) for ELISA, RNA copy number estimation, serotyping and neutralization assays with indicated virus strains. All the assays were performed once with two technical replicates and with appropriate secondary reference controls. All the assays are either internally verified or have been accredited under the ISO 17025:2017 standard.
Randomization	This is not a clinical trial, therefore, randomization is not applicable.
Blinding	This is not a clinical trial. Therefore, blinding is not applicable.

Reporting for specific materials, systems and methods

We require information from authors about some types of materials, experimental systems and methods used in many studies. Here, indicate whether each material, system or method listed is relevant to your study. If you are not sure if a list item applies to your research, read the appropriate section before selecting a response.

Materials & experimental systems

n/a	Involved in the study	
☐	☒	Antibodies
☐	☒	Eukaryotic cell lines
☒	☐	Palaeontology and archaeology
☒	☐	Animals and other organisms
☒	☐	Clinical data
☒	☐	Dual use research of concern

Methods

n/a	Involved in the study	
☒	☐	ChIP-seq
☒	☐	Flow cytometry
☒	☐	MRI-based neuroimaging

Antibodies

Antibodies used	Pan-flavivirus 4G2 anti-E antibody supernatant from D1-4G2-4-15 hybridoma cells (ATCC, Cat no: HB-112, In house Batch no: BL/CC/4G2/002), HRP-tagged anti-mouse IgG-secondary antibody (Invitrogen, Cat no: A16072, Lot no:66-110-110220), Goat anti-mouse Alexa 488 secondary antibody (Jackson ImmunoResearch, Cat No: 115545003, Lot no: 159678)
Validation	The goat anti-mouse IgG (H+L) Cross-Adsorbed Secondary Antibody, HRP (Cat. No. A16072) has been validated in WB, IHC and ELISA. The antibody has been cross-adsorbed against bovine, horse, human, pig and rabbit serum proteins and affinity purified as per the datasheet provided by the manufacturer.<br><br>For Goat anti-mouse Alexa 488 secondary antibody (Jackson, Cat No: 115545003, the specification sheet of Jackson Immuno research states as follows " Based on immunoelectrophoresis and/or ELISA, the antibody reacts with whole molecule mouse IgG. It also reacts with the light chains of other mouse immunoglobulins. No antibody was detected against non-immunoglobulin serum proteins. The antibody may cross-react with immunoglobulins from other species".<br><br>Pan-flavivirus 4G2 anti-E antibody from D1-4G2-4-15 hybridoma cells (ATCC, Cat no: HB-112) has been previously reported doi: 10.4269/ajtmh.1982.31.830

Eukaryotic cell lines

Policy information about cell lines and Sex and Gender in Research

Cell line source(s)	LLCMK2 cells (European Collection of Authenticated Cell Cultures, Cat no:85062804), D1-4G2-4-15 hybridoma cells (American Type Culture Collection, Cat no: HB-112). Baby hamster kidney cells-21 (BHK-21) (C-13) (ATCC Cat. no: CCL-10), Aedes albopictus clone C6/36 (ATCC Cat. no: CRL-1660).
Authentication	Cell lines were procured from the above sources directly for the study.
Mycoplasma contamination	All cell lines are routinely tested for mycoplasma using a commercial PCR kit (Southern Biotech Cat. No. 13100-01) and were found to be negative for mycoplasma.
Commonly misidentified lines<br>(See ICLAC register)	None
